# De Novo Characterization of Japanese Scallop *Mizuhopecten yessoensis* Transcriptome and Analysis of Its Gene Expression following Cadmium Exposure

**DOI:** 10.1371/journal.pone.0064485

**Published:** 2013-05-31

**Authors:** Xiao-lin Meng, Mei Liu, Ke-yong Jiang, Bao-jie Wang, Xue Tian, Shu-juan Sun, Zuo-yong Luo, Chu-wen Qiu, Lei Wang

**Affiliations:** 1 R&D Center of Marine, Institute of Oceanology, Chinese Academy of Sciences, Qingdao, China; 2 University of Chinese Academy of Sciences, Beijing, China; 3 College of Animal Science and Veterinary Medicine, Shanxi Agriculture University, Taigu, China; University of New England, Australia

## Abstract

**Background:**

Japanese scallop has been cultured on a large-scale in China for many years. However, serious marine pollution in recent years has resulted in considerable loss to this industry. Moreover, due to the lack of genomic resources, limited research has been carried out on this species. To facilitate the understanding at molecular level immune and stress response mechanism, an extensive transcriptomic profiling and digital gene expression (DGE) database of Japanese scallop upon cadmium exposure was carried out using the Illumina sequencing platform.

**Results:**

RNA-seq produced about 112 million sequencing reads from the tissues of adult Japanese scallops. These reads were assembled into 194,839 non-redundant sequences with open reading frame (ORF), of which 14,240 putative amino acid sequences were assigned biological function annotation and were annotated with gene ontology and eukaryotic orthologous group terms. In addition, we identified 720 genes involved in response to stimulus and 302 genes involved in immune-response pathways. Furthermore, we investigated the transcriptomic changes in the gill and digestive gland of Japanese scallops following cadmium exposure using a tag-based DGE system. A total of 7,556 and 3,002 differentially expressed genes were detected, respectively, and functionally annotated with KEGG pathway annotations.

**Conclusion:**

This study provides a comprehensive transcripts sequence resource for the Japanese scallop and presents a survey of gene expression in response to heavy metal exposure in a non-model marine invertebrate via the Illumina sequencing platform. These results may contribute to the in-depth elucidation of the molecular mechanisms involved in bivalve responses to marine pollutants.

## Introduction

The Japanese scallop, *Mizuhopecten yessoensis*, is a cold water scallop species and is distributed in northern Japan, Sakhalin, the Kuril Islands and northern Korea [1], [2]. Due to its great commercial value, this scallop was introduced to China from Japan in 1982 and expanded rapidly along the northern coastline. By 2005, the annual production of Japanese scallop had reached 150,000 tonnes in China [3].

Despite its economic importance, in recent years, *M. yessoensis* aquaculture has been hampered due to a variety of reasons such as population degradation, high temperature in summer, and other environment factors which were drastically influenced by climatic conditions and anthropogenic activities in coastal water [4], [5]. Many chemical contaminants, including organochlorine compounds, herbicides, domestic and municipal wastes, petroleum products and heavy metals are now recognized to have serious adverse effects on aquaculture environments, even when released at low levels [6], [7]. However, the impact of these pollutants on *M. yessoensis* is unclear, especially at the molecular level. Previous related studies on *M. yessoensis* have mainly focused on testing bioaccumulation in tissues [8], [9], effects on antioxidant enzyme activity [4], [10], lipid peroxidation [11], [12], DNA strand break [13], [14], the discovery of genetic markers [15]–[18] and the construction of gene maps [19]. Nevertheless, due to the lack of genomic resources such as genome and transcriptome sequences, these studies were limited and an overall understanding of the mechanism of action of these pollutants in *M. yessoensis* is a high priority. To achieve this, a global understanding of the transcriptome profiling of *M. yessoensis* is the first and necessary step.

The massive parallel next generation sequencing technologies have facilitated the production of high coverage sequence data, enabling genome wide assays of transcriptional activities and have been applied in many types of aquatic animals such as zebrafish [20], carp [21], sea bass [22], and clam [23]. Although the 454 and SOLID platforms have their own superiority, recent algorithmic and experimental advances have greatly increased the applicability of Illumina sequencing and *de novo* assembly, which has been successfully and increasingly used for non-model species [22], [24]–[26]. In addition, Illumina sequencing technology has been shown to be highly replicable and was proven to be a superior method to study mRNA expression levels due to its ability to identify differentially expressed genes [27]. To our knowledge, this is the first report on the transcriptome profiling of adult *M. yessoensis* using the Illumina facility with the aim of constructing a database on this species.

Cadmium pollution is a serious problem in Bohai Bay, China, according to an ecological assessment conducted from 2001–2005 [28]. Therefore, understanding its toxicological mechanism and effective early warning of aquaculture risk in *M. yessoensis,* according to the biomarker monitoring system, is now necessary. In this study, digital gene expression (DGE) technology was applied to analyze the differential expression of genes in the gill and digestive gland of *M. yessoensis* following cadmium (Cd) exposure, and a sensitive biomarker database was constructed. Our results could provide valuable and reliable data for bivalve aquaculture and are expected to improve our understanding of the toxicological mechanism of marine pollutants.

## Results and Discussion

### Illumina Paired-end Sequencing and Reads Assembly

**Figure 1:**
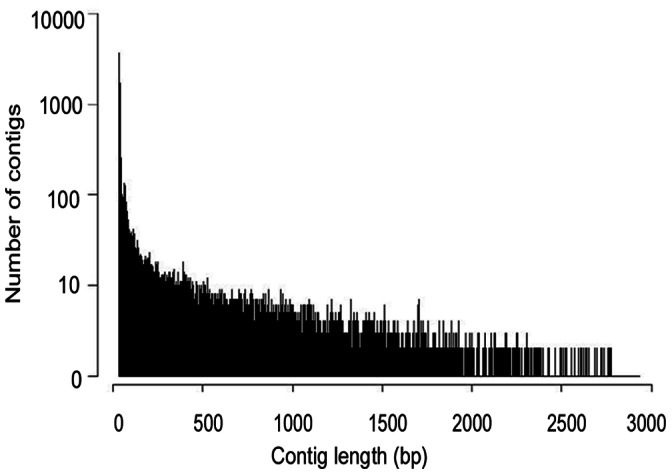
Assembled contig length distribution of the *M. *yessoensis transcriptome. The x-axis indicates contig size and the y-axis indicates the number of contigs of each size.

To obtain a global overview of *M. yessoensis* transcriptome and gene activity at nucleotide resolution, a cDNA pool was prepared from seven organs including the adductor muscle, digestive gland, gill, gonad (male and female), kidney, visceral mass and mantle and sequenced using the Illumina sequencing platform. Approximately 112.26 million 93 bp reads were generated. The raw reads produced in this study have been deposited in the NCBI SRA database (accession number: SRR653778). After removing adaptors and low quality reads, reliable reads were *de novo* assembled with Velvet and Oases software. A total of 217,190 contigs were assembled ranging from 100 to 29,088 bp, with an average size of 436 bp. Among these contigs, 184,390 (∼84.89%) of the clean contigs were smaller than 500 bp, 12,703 (5.85%) were between 500 and 1,000 bp, 13,847 (∼6.38%) were between 1,000–3,000 bp in length, and 6,250 (∼2.88%) were longer than 3,000 bp. The length distribution of assembled contigs was shown in Figure 1. The previously reported *M. yessoensis* transcriptome by 454 sequencing generated 32,590 contigs with a mean size of 618 bp [29],which is 180 bp longer than the length in our current study, yet we obtained more detailed data in terms of quantity of the database. After the gene prediction program, a total of 194,839 contigs (with an average length of 461 bp) from these non-redundant sequences were found including putative open reading frames (ORF) which encoded proteins using the GetORF program of the EMBOSS suite. A summary of the data was shown in Table 1.

**Table 1:** Summary of Illumina transcriptome sequencing and assembly for *M. yessoensis.*

Total number of reads	112,265,296
Total base pairs (bp)	10,440,672,528
Average read length (bp)	93
Total number of non-redundant contigs	217,190
Mean length of non-redundant contigs (bp)	436
Contigs with coding protein sequences	194,839
Mean length of contigs with coding protein sequences (bp)	461

To demonstrate the quality of the sequencing data, 6 contigs were selected and 6 pairs of primers were designed for RT-PCR amplification. Agarose gel electrophoresis results showed that 5 of the 6 primer pairs obtained a band of the expected size, and the five identified PCR products were further confirmed by Sanger sequencing (data not shown).

### Annotation of Predicted Proteins

**Figure 2:**
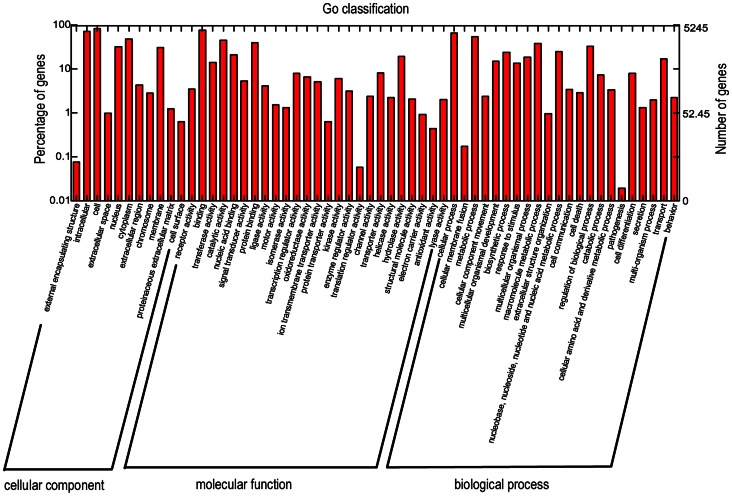
GO annotations of *M. yessoensis* transcriptome data. A total of 5,249 contigs are assigned to at least one GO term including biological process, cellular component and molecular function.

**Figure 3:**
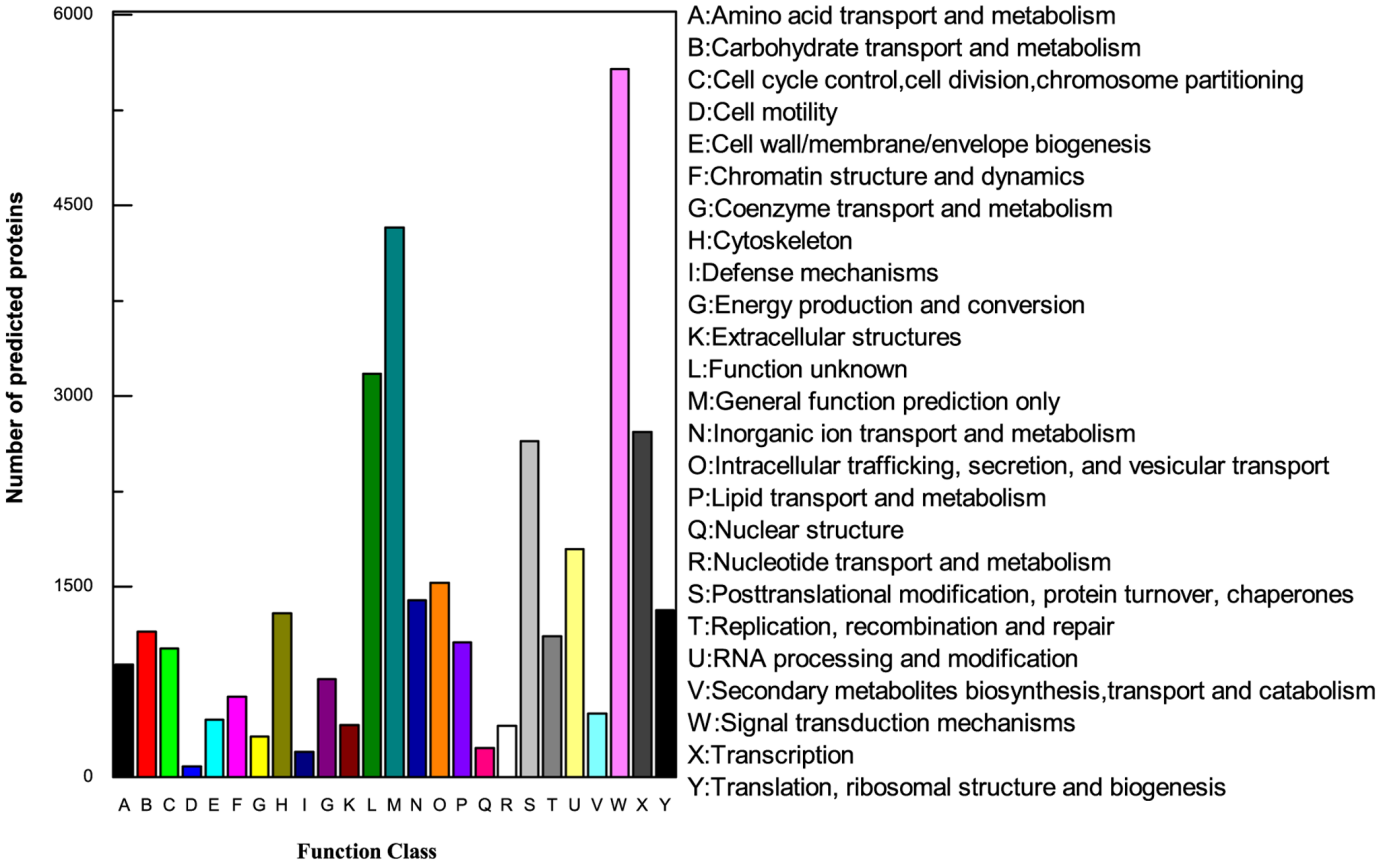
KOG annotations of predicted proteins. A total of 34,839 predicted proteins have a KOG classification among the 25 categories.

All the predicted protein sequences (translation by the Transeq program of the EMBOSS suite) were compared with the NCBI non-redundant (nr) protein database and the Swiss-Prot database for gene annotation using BLASTx with an E-value cut-off of le-3. A total of 14,240 predicted protein sequences obtained biological function annotation, while the remainder would require more genetic data for annotation in future. Gene ontology (GO) assignments were used to classify the functions of the predicted proteins. Based on GoPipe software, a total of 5,245 predicted proteins were assigned at least one GO term for describing cellular components, molecular functions and biological process (Figure 2). Briefly, in the cellular components category, cell (30.3%) was the dominant group followed by intracellular (25.8%). For molecular functions, binding (27.5%) was the most representative GO term, followed by catalytic activity (16.2%). With regard to biological process, the most represented categories were cellular process (19.5%) and metabolic process (16.2%) (Figure 2). These results corresponded with the previously sequenced *M. yessoensis* via 454 sequencing [29]. To further evaluate the completeness of the transcriptome library and the effectiveness of the annotation process, eukaryotic orthologous groups (KOG) were used. In total, 34,989 annotated putative proteins were classified functionally into 25 molecular families, including signal transduction mechanism, cellular structure, biochemical metabolism, defence system, gene expression and so on (Figure 3).

Besides GO annotation and KOG analysis, we mapped all the predicted proteins to the reference canonical pathways in KEGG (Kyoto Encyclopedia of Genes and Genomes) [30] for functional categorization and annotation. In total, we assigned 2,702 proteins into different KEGG pathways and the most representative pathways were infectious diseases (678 members), cancers (482 members), signal transduction (438 members) and carbohydrate metabolism (352 members) (Table 2). These well-categorized and annotated resources could provide an important and valuable database for investigating specific bioprocesses, identification of functional genes and analyzing specific traits during *M. yessoensis* research.

**Table 2:** KEGG biochemical mappings for *M. yessoensis.*

KEGG categories represented	Unique sequences (The number of enzymes)
**Metabolism**	
Carbohydrate Metabolism	352 (172)
Energy Metabolism	117 (96)
Lipid Metabolism	308 (166)
Nucleotide Metabolism	184 (112)
Amino Acid Metabolism	301 (169)
Metabolism of Other Amino Acids	102 (61)
Glycan Biosynthesis and Metabolism	226 (124)
Metabolism of Cofactors and Vitamins	133 (110)
Metabolism of Terpenoids and Polyketides	32 (24)
Biosynthesis of Other Secondary Metabolites	43 (23)
Xenobiotic Biodegradation and Metabolism	142 (58)
**Genetic Information Processing**	
Transcription	144 (135)
Translation	278 (227)
Folding, Sorting and Degradation	321 (285)
Replication and Repair	202 (122)
**Membrane Transport**	
ABC transporters	27 (24)
Signal Transduction	438 (261)
Signal Molecules and Interaction	86 (73)
**Cellular Process**	
Transport and Catabolism	293 (241)
Cell Motility	59 (56)
Cell Growth and Death	317 (154)
Cell Communication	156 (120)
**Organismal systems**	
Immune system	302 (155)
Endocrine System	242 (152)
Circulatory System	47 (36)
Digestive System	194 (97)
Excretory System	70 (58)
Nervous System	282 (131)
Sensory System	32 (18)
Development	76 (68)
Environmental Adaptation	25 (19)
**Human Diseases**	
Cancers	482 (166)
Immune Diseases	38 (31)
Neurodegenerative Diseases	201 (119)
Cardiovascular Diseases	52 (28)
Endocrine and Metabolism Diseases	33 (28)
Infectious Diseases	678 (304)

### Functional Genes Involved in Environmental Stress and Immunity

In many bivalve species, responses to environmental stress and changes in immunity are used as biomarkers to evaluate the biological effects of different types of pollutants in aquatic animals, including metallothionein (MT), lysosomal membrane integrity, acetylcholinesterase (AChE), the cytochrome P450 (CYP) family, glutathione-dependant oxidoreductases and heat shock proteins (HSP) [31]–[36]. In addition, due to a lack of immunoglobulins, invertebrates have developed unique systems of biological host defense, the so called innate immunity [37]. However, the exact molecular and cellular basis of the immune system remains poorly understood. In this study, the sequence and annotation information from BLAST, GO and KEGG annotations provided valuable gene resources for research on the molecular biology of these traits in *M. yessoensis*.

The GO annotation identified 720 sequences that potentially respond to stimulus (GO: 0050896) (Table S1). DNA damage in marine organisms, one of the major effects following the pollutants stimulus, may be relevant to DNA strands broken which can be induced by free radicals, organic and inorganic contaminants, heavy metals, etc. Therefore, it is essential for cells to efficiently respond to DNA damage through coordinated and integrated DNA-damage checkpoints and repair pathways. However, the mechanism of the DNA damage repair in bivalves is not very clear. The DNA repair protein family, DNA mismatch repair protein family and DNA excision repair protein family [38]–[40] which play an important role in DNA damage repair after challenge were presented abundant in this category. These data could be valuable for future research. HSP have a long history of use in studies of organism stress response. In this term of GO annotation, the HSP families including HSP70, HSP90, HSP110 and HSP transcription factor were detected which play essential roles as molecular chaperones by assisting the correct folding of nascent and stress-accumulated misfolded proteins and by preventing their aggregation [36], but others, such as HSP27, HSP40, HSP10, HSP22 were not detected. In addition, other genes which have been used as biomarkers in environmental monitoring were detected, such as glutathione S-transferases (GST), Glutathione peroxidase (GPx), CYP family, SOD, and CAT. It is worth noting that the HSP families were the most abundant transcripts in this category in Hou’s report [29] and the stress-associated endoplasmic reticulum protein (SERP2) was also highly expressed, which is different from our data, due possibly to the different samples used in the two experiments.

Although knowledge of bivalve immune-related genes has increased in the last few years with the development of biotechnology, the available information is still scarce and fragmentary [41]. With regard to the immune system, KEGG analysis resulted in 251 genes involved in 13 immune-response pathways (Table 2, Table S1) such as Fc gamma R-mediated phagocytosis signaling pathway, chemokine signaling pathway, complement and coagulation cascades, Toll-like receptor signaling pathway and NOD-like receptor signaling pathway. The complement system consists of a tightly regulated network of proteins that play crucial role in host defense and inflammation. C3 and C1 q are the central components in this system and were detected in this database. Although the complement pathway has not been extensively described in bivalves, our results support the presence of this defense mechanism. Toll-like receptors (TLRs) are an ancient family of pattern recognition receptors that play key roles in detecting non-self substances and activating the immune system. TLR1, TLR 4, MYD88, TRAF and IRAK, were identified in the database, which have been established to play an essential role in the activation of innate immunity by recognizing specific patterns of microbial components [42]. Tumor necrosis factor receptor-associated factor 3 (TRAF3), a highly versatile regulator, played important roles beyond the TNFR-superfamily (SF), mediating certain innate immune receptor and cytokine receptor signals [43]. ESTs with homology to the TRAF3 have been detected in the pearl oyster [44] and were also presented in the RIG-I-like receptors (RLRs) and TLRs signaling pathways. PI3 K [45], MAPK [46], and p38 [47], which are also crucial in cellular responses to external stress signals, were found in corresponding pathways. Therefore, the large set of immune-relevant genes and their roles in responses to environmental stress may significantly improve our understanding of the immune mechanism in *M. yessoensis*.

### Digital Gene Expression Profile Analysis

**Figure 4:**
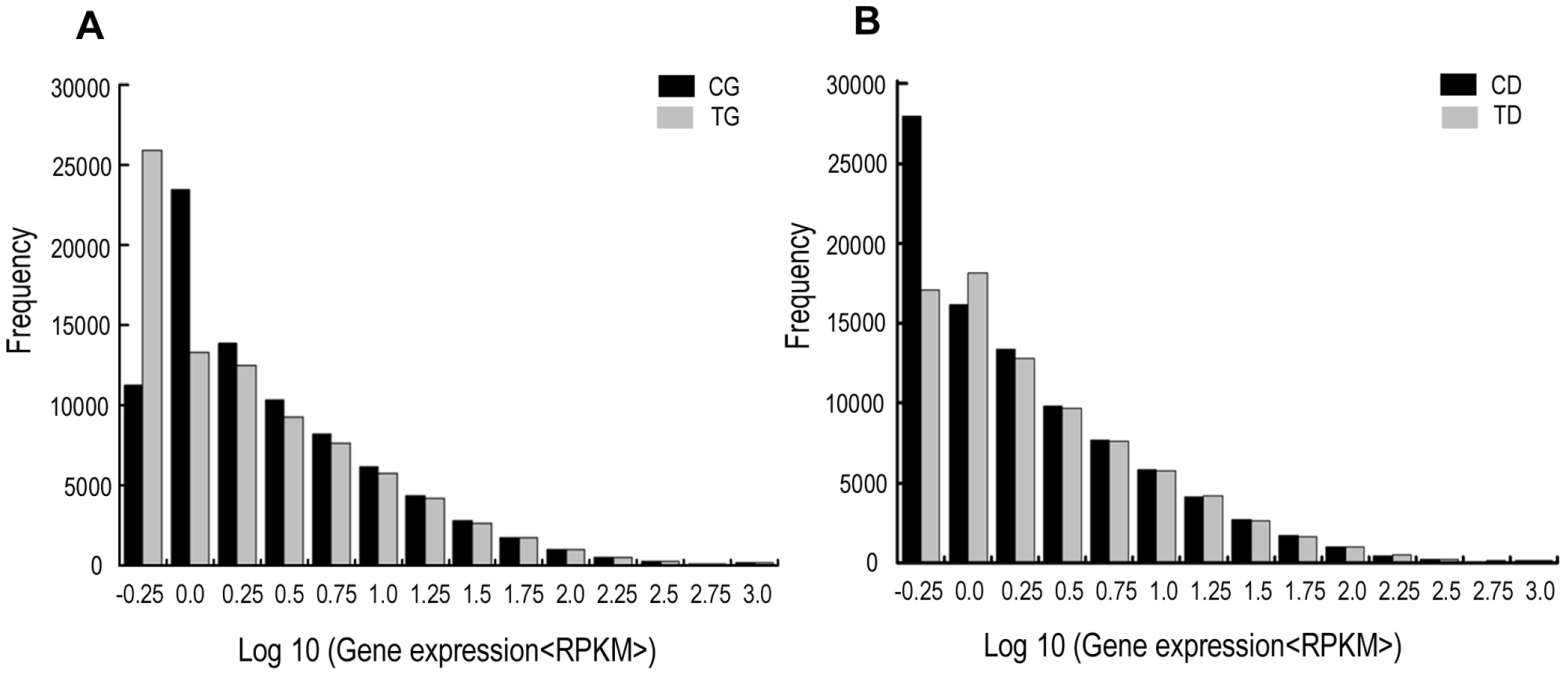
The level of gene expression for each gene. Gene expression level was determined by calculating the number of reads for each gene and then normalized to RPKM (Reads per kilobase per Million). A indicates the distribution of genes in CG and TG, B indicates the distribution of genes in CD and TD.

DGE analysis based on the Solexa/Illumina system, which avoids many of the inherent limitations of microarray analysis, was performed to identify the genes involved in the response of *M. yessoensis* to cadmium (Cd) exposure. We sequenced four DGE libraries: gill in the control (CG), digestive gland in the control (CD), gill in the treated group (TG) and digestive gland in the treated group (TD), and generated 5.75, 9.21, 8.96 and 6.95 million raw reads, respectively (Table 3). After removing the low quality reads, the number of clean reads ranged from 5.67 to 9.12 million (98.75–99.0%). The data sets are available at the NCBI SRA with the accession number: SRR654701. Gene annotation was performed by tag mapping analysis using the 217,190 non-redundant sequences from RNA-seq based transcriptome analysis as the reference transcript database. Among the clean reads generated by Illumina sequencing of the four libraries, about 1.86 million to 2.95 million sequences were mapped to the entire reference database (Table 3). The level of gene expression was analyzed by calculating the number of mapped reads for each gene locus and then normalizing it to the number of Reads per Kilobase per Million (RPKM) [48]. As shown in Figure 4, the results indicated that the major types of expressed genes were represented in fewer than 18 copies and a small percentage of the genes were highly expressed.

**Table 3:** Statistics of DGE sequencing.

Summary	CG	TG	CD	TD
Raw reads	5,745,741	8,958,064	9,210,131	6,946,099
Clean reads	5,674,105	8,864,949	9,118,668	6,873,165
Clean reads (%)	98.75%	98.96%	99.00%	98.95%
Low quality (%)	1.25%	1.04%	1.00%	1.05%
Reads mapped to genes	1,856,721	2,865,585	2,951,986	2,198,404
Mapped rate (%)	32.72%	32.32%	32.37%	31.99%

### Changes in Gene Expression in the Gill and Digestive Gland Following Cd Exposure

To calculate the expression abundance of each gene between CG and TG or CD and TD, the MARS model [49] in the DEGseq package was used. A total of 7,556 significantly changed gene entities were observed between CG and TG corresponding to 3,673 genes up-regulated and 3,883 genes down-regulated (Table S2). Between CD and TD, a total of 3,002 differentially expressed genes were detected with 1,627 up-regulated genes and 1,375 down-regulated genes (Table S3). This suggested that the number of differentially expressed genes between CG and TG was more than those between CD and TD. The top 20 most differentially expressed genes between samples (TG vs CG; TD vs CD) were then analyzed (Table S4). We observed that most of the highly expressed genes in both of the compared groups were orphan sequences which meant that no homologues were found in the Nr database. This might be due to a lack of clear annotation information on molluscs in the database and the function of these unknown genes needs to be identified in the future. To identify more realistic different expressed genes, negative binomial distribution was introduced [50]and we obtained 70 up- and 47 down-regulated genes in gill and 81 up- and 43 down-regulated genes in digestive gland (Table S5).

### Function Annotation of Differentially Expressed Genes

To understand the functions of differentially expressed genes (DEGs), we mapped all the genes with expression abundance more than 2-fold to terms in the KEGG database and compared this with the whole transcriptome background. Among all the genes with KEGG annotation, 479 differentially expressed genes were observed between CG and TG (Table S6). Most of them were involved in proteasome, ribosome biogenesis in eukaryotes, ATP-binding cassette (ABC) transporters, MAPK signaling pathway and oxidative phosphorylation. Between CD and TD, 154 differentially expressed genes were annotated with KEGG pathway annotation (Table S6). The main enriched metabolism pathways concentrated on ABC transporters, glycine, serine and threonine metabolism, steroid hormone biosynthesis and glutathione metabolism. Cellular process including lysosome and peroxisome were also enriched.

Global cellular stress response has been defined as all proteins over-produced due to environmental stress. Following Cd exposure, several protein families including ABC, HSP and CYP were observed over transcribed in gill or digestive gland of *M. yessoensis*. Previous work has indicated that ABCB- and ABCC-type transporters confer multixenobiotic resistance and form an environment-tissue barrier in bivalve gills [51]. This protective mechanism is referred to as multixenobitic resistance (MXR) which is supposedly ubiquitous in aquatic invertebrates. Also, other research suggests that the ABC multidrug transporters could be act as a detoxifying mechanism of various toxic agents in aquatic organism [52]. This might be the reason that ABC families also found significantly up-regulated in digestive gland in our results. In addition to ABCB and ABCC, ABCA, ABCD and ABCG were detected simultaneously up-regulated. As we discussed previously, HSP families are critical factors during the process of environmental stress. The present study found that HSP70, HSP110 in gill and HSP70 in digestive gland were over transcribed.

Comparing the enriched different expressed genes between gill and digestive gland following Cd exposure, several interesting pathways and genes were explored. NADH dehydrogenase, ATPase and cytochrome c oxidase of oxidative phosphorylation were only induced in gill of *M. yessoenis* by Cd, suggesting a compensation of partial uncoupling of oxidative phosphorylation [53] and the key role of the gill in energy metabolism. DNA damage and DNA damage repair were the major effects of pollutants stimulus. Related DNA-damage-inducible protein and DNA mismatch repair protein were also detected up-regulated only in gill. This probably related with the function of gill which acts as the main tissue for filtration of suspended matter and forms the first defense barrier.

Genes encoding detoxification enzymes played important role in bivalves when exposed to chemical pollutants and related evidences in previous study have indicated that digestive gland was responsible for detoxification of environmental pollutants and suggested it as a target organ for detection/identification of biomarkers of pollutants [23]. These detoxification enzymes include CYP families, GST, AChE and so on. CYP are one of the major phase I-type classes of detoxification enzymes found in terrestrial and aquatic organisms ranging from bacteria to vertebrates [54]. In our pathway enrichment analysis to digestive gland, totally 9 transcripts of cytochrome P450 families were found and eight of them presented up-regulate including CYP17, CYP1A1, CYP3A, CYP5A, CYP4F, CYP26A, CYP98A. Although CYP also be detected in gill, the highest expression was observed in digestive gland. GSTs constitute a large protein family, with a pivotal role in detoxification of xeno-compounds [23]. An increased GST activity has been observed after exposure to a broad set of pollutants. Following Cd exposure, two transcripts encoding GST were detected over-expressed in digestive gland in pathway of glutathione metabolism. However, the same tendency was not observed in gill. It could be due to the tissue-specific expression and sensitivity to dose/type of chemicals of GST in bivalves. At the cellular level, loss of lysosomal membrane integrity has been observed as a consequence of oxidative stress induced by several classes of chemicals. Reduced lysosomal membrane stability is also linked to increased autophagy [23], [55]. Therefore, Cd exposure may induce lysosomal damage in the digestive gland of *M. yessoensis*.

### Experimental Validation of Differentially Expressed Genes

To further evaluate the DGE library, nine genes expressed in the gill or digestive gland with clear annotation (2 up-regulated, 2 down-regulated and 2 controls for each tissue) were randomly selected for analysis by qRT-PCR. The qRT-PCR results displayed the same expression tendency as the DGE library (Table 4). Interestingly, yellow –x1c (Y-x1c) which was significantly down-regulated in the gill was highly expressed in the digestive gland. However, yellow-like genes have only been identified in insect species and in a number of bacteria [56]. The function of yellow-like genes is largely unknown and requires further research. GPx, often used as a biomarker of environmental stimulus, was significantly down-regulated in the gill. This finding is consistent with several previous studies [57], [58] which directly tested the enzyme activity using a biochemical test. In contrast, CYP, another biomarker used in monitoring environmental contamination [34], was down-regulated in the digestive gland, but displayed different expression in other studies [59], [60], probably due to different species, tissues or exposure time and dosage. GTPase and IMAP family 1 (GIMAP1), believed to be a regulator of cell death [61], were down-regulated in the digestive gland, which may mean immunologic mjury and needs to be identified by other ways. SPF27 and scramblase were used as controls and did not show a difference in expression in gill and digestive gland tissues. These findings provide us with more valuable molecular targets for the in-depth study of oxidative damage and immunologic injury in *M. yessoensis*.

**Table 4:** Validation of DGE library by qRT-PCR.

Gene	Gene ID	DGE library	fold by DGE	fold by qPCR
*Hzf3*	locus_34761	TG vs. CG	3.48	4.24
*Nephrin*	locus_134559	TG vs. CG	7.25	10.16
*Y-x1c*	locus_22146	TG vs. CG	−10.52	−18.26
	locus_4770	TD vs. CD	7.37	6.37
*Gpx*	locus_16910	TG vs. CG	−4.14	−2.18
*Hsp70*	locus_20269	TD vs. CD	2.27	2.63
*CYP*	locus_14839	TD vs. CD	−5.77	−2.85
*GIMAP1*	locus_20540	TD vs.CD	−5.40	−6.33
*SPF27*	locus_100	TG vs. CG	−0.86	/
	locus_100	TD vs.CD	−0.49	/
*Scramblase*	locus_10003	TG vs. CG	0.23	/
	locus_10003	TD vs. CD	0.89	/

### Conclusions

In this study, we generated a comprehensive transcriptome of adult *M. yessoensis* using the Illumina sequencing platform. A single run produced 194,839 distinct sequences with 14,240 sequences having explicit annotation. A significant number of genes involved in immunity and response to stimulus were found in this transcriptome. These findings substantially supplement existing sequence resources for *M. yessoensis*. To our knowledge, this is the first publication using Illumina sequencing technology in this species, which lack a reference genome. In addition, genes differentially expressed in the gill and digestive gland following cadmium exposure were identified and functionally annotated with the KEGG database. These data would provide potential molecular targets in shellfish for functional studies of genes responding to marine pollutants and would serve as a valuable reference for identifying biomarkers in marine environmental pollution monitoring.

## Materials and Methods

### Experimental Scallop

Adult Japanese scallops (*Mizuhopencten yessoensis*) of both sexes were collected from Dalian Zhangzidao (Dalian, Liaoning Province, China) in 2011. Housing and care of scallops and collection of tissue samples for use in described experiments were conducted in accordance with the International Guiding Principles for Biomedical Research Involving Animals (http://www.cioms.ch/frame 1985 texts of guidelines. html).Only healthy scallops of a homogeneous size (8.730±0.167 cm (mean±SD, n = 30), shell height) were used. They were kept in running aerated sea water (salinity 30‰) at 16±1°C which was pumped from the first bathing beach of Qingdao and filtered prior to the experiments. Half the seawater was changed daily and the scallops were fed with the microalgae *Spirulina*.

### Cadmium Exposure and RNA Preparation

The scallops in the experimental groups were exposed to CdCl_2_·2.5 H_2_O (Kanto Chemical Co., Tokyo, Japan) and the final Cd^2+^ concentration in the seawater was 0.1 mg/L which was twenty times that of the standard according to the ‘Water quality standard for fisheries of China’ (Cd^2+^≤0.005 mg/L). Three replicates were prepared for both the control and Cd treated group. They were all kept under the same conditions described above and the changed seawater was resupplied with the corresponding concentration of Cd. No mortality was observed during the experimental period. After 14 days exposure, tissues including the adductor muscle, digestive gland, gill, gonad (male and female), kidney, visceral mass and mantle in the control were collected for RNA-seq. In addition, the gill and digestive gland in the control or treated groups were collected, respectively, for DGE analysis. At least three independent biological replicates for each sample were harvested. Total RNA was isolated using TRIzol reagent (Gibco BRL) following the manufacturer’s protocol. The quantity and quality of total RNA was confirmed using a NanoDrop spectrophotometer (Thermo Scientific, USA) and an Agilent 2100 Bioanalyzer (Agilent Technologies). It was then treated with DNase I (Ambion, USA) for 1 h at 37°C to remove residual DNA.

### cDNA Library Preparation and Illumina Sequencing for RNA-seq

One paired-end (PE) cDNA library was generated from the pooled total RNA of the adductor muscle, digestive gland, gill, gonad (male and female), kidney, visceral mass and mantle in equal quantity. In brief, oligo (dT) beads were used to isolate poly (A) mRNA (mixture). First-strand cDNA was synthesized using a random hexamer-primer and reverse transcriptase (Invitrogen). Second-strand cDNA was synthesized using RNase H (Invitrogen) and DNA polymerase I (TaKaRa, Japan). The cDNA was sonicated for 5 min using the cup horn Sonic Dismembrator 550 (Fisher Scientific). The fragmented library was fractionated on 8% polyacrylamide gels, and 300–500 bp base pair fractions were excised. The gel purified cDNA product was modified for sequencing using the TruSeq™ DNA Sample Prep Kit- Set A (Illumina, USA) and then PCR-amplified with TruSeq PE Cluster Kit (Illumina, USA).

### Analysis of Illumina Sequencing Results

The cDNA library was sequenced on the Illumina sequencing platform (GA II) and raw PE reads with an average length of 93 bp were generated. The raw reads were then assembled into the non-redundant consensus using Velvet (version 0.7.62; http://www.ebi.ac.uk/~zerbino/velvet/), which was developed for assembly of short reads using the de Bruijin graph algorithm [62]. In addition, another publicly available program, Oases (version 0.1.8; http://www.ebi.ac.uk/~zerbino/oases/) was used, which was also developed for *de novo* assembly of short reads in order to obtain best assembly results [63]. Adapter sequences were then trimmed and low quality reads were removed. Short sequences (<100 bp in length) were also removed using the custom Perl Program [64]. Putative open reading frames (ORF) within contigs were then obtained which encoded proteins using the GetORF program of the EMBOSS suite [65]. The ORF of each predicted protein was then used for BLASTx searches against the NCBI non-redundant (nr) protein database and Swiss-Prot protein database with an E-value threshold to 10^−3^. Functional annotation using gene ontology terms (GO; http://www.geneontology.org) was analyzed using the BLASTp algorithm against the Swiss Prot and TrEMBL databases by the GoPipe program according to gene2go software [66] at http://gopipe.fishgenome.org). The COG and KEGG pathways annotation was performed using Blastall software against the Cluster of Orthologous Groups database and Kyoto Encyclopedia of Genes and Genomes database [67].

### Digital Gene Expression (DGE) Library Preparation and Sequencing

Four tag libraries from the control gill (CG), the control digestive gland (CD), the treated group gill (TG) and the treated group digestive gland (TD) were prepared for DGE which was performed in parallel using the Digital Gene Expression Tag Profile Kit (Illumina). Briefly, total RNA from the four samples was enriched using oligo-dT magnetic beads. The first and second cDNA strands were synthesized and bead bound cDNA was subsequently digested with *Nla*III. The 3^′^-cDNA fragments were then purified with oligo(dT) beads and 5′ ends were added with Illumina adapter 1, which contained a recognition site for the endonuclease *Mme*1 for cutting 17 bp downstream of the recognition site (CATG) to produce tags with adapter 1. After removing 3′ fragments with magnetic bead precipitation, an Illumina GEX adapter 2 was introduced at 3′ ends of the tags. The resulting cDNA tags with adapter-ligated were amplified using PCR amplification for about 15 cycles. The four enriched cDNA tag libraries were then gel-purified and checked using the Agilent 2100 Bioanalyzer. Each cDNA tag library was digested and the single-chain molecules were fixed onto an Illumina proprietary sequencing chip (flow cell) and underwent deep sequencing using the Illumina Genome Analyzer.

### Aligning DGE Tags to the Reference Transcriptome

Raw sequencing reads were filtered by the Illumina pipeline. Prior to mapping reads to the reference transcriptome generated by RNA-seq, all low quality sequences such as short sequences (<21 nt), empty reads, and singletons (tags that occurred only once) were filtered. To monitor mapping events on both strands, both sense and antisense sequences were included in the data process. Only perfect matches of 17-bp tags plus the CATG *Nla*III recognition site were allowed.

### Analysis of Differentially Expressed Genes

For gene expression analysis, the number of expression tags from the four samples was calculated and normalized to Reads per Kilobase per Million (RPKM) [48], respectively. Then the expression abundance of each gene between the control and treated group (gill or digestive gland) was counted based on the MARS model (MA-plot-based method with Random Sampling model) [49] using the DEGseq package. In this study, we used stringent value FDR (false discovery rate) <0.001 and the absolute value of |log_2_^Ratio^|≤1 as the threshold to judge the significant difference in gene expression. In order to get more realistic data, the algorithm based on negative binomial distribution was implemented [50]. For pathway enrichment analysis, all differentially expressed genes were mapped to terms of the KEGG database and significantly enriched KEGG terms were identified.

### Quantitative Real-time PCR (qRT-PCR) Verification

Nine genes were selected for confirmation of DGE data by qRT-PCR using the SYBR Premix Ex Taq kit (Takara, Japan) according to the manufacturer’s instructions and performed in triplicate on the stratagene Mx3005P system. Briefly, total RNA was isolated from the gill and digestive gland of *M. yessoensis* in the control and Cd treated group (at least 4 scallops for each sample), using TRIzol reagent (Invitrogen, USA). First strand cDNA was obtained from 2 µg of total RNA using the PrimeScript II 1st strand cDNA synthesis kit (Takara, Japan). The specific primers used for qRT-PCR are listed in Table S7 and β-actin was used as an endogenous control. The relative gene expression was analyzed using the comparative threshold cycle (CT) method established by Livak et al. [68].

## Supporting Information

Table S1 Genes involved in environmental stress and immunity. A list of the potential genes involved in environmental stress and immunity based on BLAST, GO and KEGG annotation.
(XLSX)

Table S2 Different expressed genes between CG and TG. A list of the different expressed genes in the gill of *M. yessoensis* after Cd exposure. FDR (<0.001) and P-value (<0.001) were used as the standard.
(XLSX)

Table S3 Different expressed genes between CD and TD. A list of the different expressed genes in the digestive gland of *M. yessoensis* after Cd exposure. FDR (<0.001) and P-value (<0.001) were used as the standard.
(XLSX)

Table S4 The top 20 differentially expressed genes between CG and TG or CD and TD. The top 20 differentially expressed genes after Cd exposure in the gill and digestive gland were analyzed.
(XLSX)

Table S5 Different expressed genes between CG and TG or CD and TD. A list of different expressed genes based on negative binomial distribution.
(XLSX)

Table S6 Pathway enrichment analysis of different expressed genes. A list of pathway enrichment analysis of different expressed genes between TG and CG or TD and CD.
(XLSX)

Table S7 Primers for qRT-PCR validation.
(XLSX)
